# Improving Efficiency and Quality of Handover in the Mental Health Liaison Team (MHLT): A Focus on Achieving Team Buy-In

**DOI:** 10.1192/bjo.2022.345

**Published:** 2022-06-20

**Authors:** Bharat Velani, Sagar Jobanputra, Chiemezie Ukachukwu, Adeagbo Osundina, Niranga Karunaratne, Tanya Deb

**Affiliations:** Hertfordshire Partnership University NHS Foundation Trust, London, United Kingdom

## Abstract

**Aims:**

To Reduced Mental Health Liaison Team (MHLT) handover time to less than 30 minutes within one month and to improve the quality of handover. The non-medical staff have been part of the team for many years, whilst medical staff have recently changed or are on short rotations. Previous changes have not been well sustained. Much of the initial enthusiasm for this project was coming from the medical staff members. We felt that it was important to fully explore the driving human factors to achieve sustainable buy-in.

**Methods:**

The total period of the project was 7 weeks. First two weeks were used for daily baseline data-collection and informal and formal discussions with team members to formulate driver diagram and change ideas. Two “Plan, Do, Study, Act” (PDSA) cycles with two intervention points at week 3 and week 4.

**Results:**

Key human factors identified in the MHLT were burnout and emotional fatigue, core team values (cohesion, flexibility, and camaraderie), and disillusion with authority and imposed change. Contributing factors to burnout and emotional fatigue were long and short-term staff sickness, chronic under-staffing, and systemic changes in the general hospital due to the COVID-19 pandemic. The human factors were used to guide key decisions in methodology and creation of change ideas. These decisions included: Avoidance of surveys and questionnaires (staff request), limiting the total number of changes, any additional administration to be undertaken by medical staff, and avoiding a rigid handover system. Following 2 PDSA cycles, there were improvements in average length of handover from 44 minutes (2-week baseline data) to 30 minutes (4-weeks post second intervention). When compared to the baseline data there were also improvements in the average number of interruptions (7 vs 2), availability of key information (69% vs 92%), allocation of staff member (80% vs 95%) and allocation of review date (83% vs 95%). No difference in the average number of patients for handover discussion between 2-week baseline data (15) and the 5 weeks after (15).

**Conclusion:**

The aims for the Quality Improvement Project were met and a plan has been set to re-audit in both 6 months and 1 years’ time to test sustainability of change. Sudden illness and effects of the COVID-19 pandemic have led to short and long-term staff shortage, contributing to burnout and emotional fatigue. Attention to the unique human factors involved in team dynamics and staff morale can help achieve buy-in and real change.

